# Who is more susceptible to job stressors and resources? Sensory-processing sensitivity as a personal resource and vulnerability factor

**DOI:** 10.1371/journal.pone.0225103

**Published:** 2019-11-18

**Authors:** Tinne Vander Elst, Maarten Sercu, Anja Van den Broeck, Elke Van Hoof, Elfi Baillien, Lode Godderis

**Affiliations:** 1 Knowledge, Information and Research Center, IDEWE Group (an External Service for Prevention and Protection at work), Leuven, Belgium; 2 Research Group Work, Organisational and Personnel Psychology, KU Leuven, Leuven, Belgium; 3 Research Group of Work and Organizational Psychology, Vrije Universiteit Brussel, Brussels, Belgium; 4 Research Centre for Work and Organisation Studies, KU Leuven, Brussels, Belgium; 5 Optentia, North West University, Vanderbijlpark, South Africa; 6 Departement Psychology (PSYCH), Faculty of Psychological and Educational Sciences, Vrije Universiteit Brussel, Brussels, Belgium; 7 Department of Psychosocial Science, University of Bergen, Bergen, Norway; 8 Environment and Health, KU Leuven, Leuven, Belgium; Universitat de Valencia, SPAIN

## Abstract

This study aimed to investigate whether people scoring higher (compared to lower) on sensory-processing sensitivity respond differently to the work environment. Specifically, based on the literature on sensory-processing sensitivity and the Job Demands-Resources model, we predicted that the three components of sensory-processing sensitivity (i.e. ease of excitation, aesthetic sensitivity and low sensory threshold) amplify the relationship between job demands (i.e. workload and emotional demands) and emotional exhaustion as well as the relationship between job resources (i.e. task autonomy and social support) and helping behaviour. Survey data from 1019 Belgian employees were analysed using structural equation modelling analysis. The results showed that ease of excitation and low sensory threshold amplified the relationship between job demands and emotional exhaustion. Low sensory threshold also strengthened the job resources–helping behaviour relationship. This study offered first evidence on the greater susceptibility among highly sensitive persons to the work environment and demonstrated that the moderating role might differ for the three components of sensory-processing sensitivity. Additionally, it adds sensory-processing sensitivity to the Job Demands-Resources model and highlights the idea that personal factors may act both as a personal vulnerability factor and a personal resource, depending on the nature of the perceived work environment.

## Introduction

Sensory-processing sensitivity (SPS)—a trait characterised by being more sensitive to the environment—has been identified in more than 100 species [1]. It may manifest psychologically (e.g., greater emotional reactivity and low sensory screening; [1]), physiologically (e.g., increased brain activation when making fine visual distinctions; [2]), as well as genetically (e.g., SPS has been connected to the 5-HTTLPR s/s genotype responsible for transporting serotonin; [3]). The concept of SPS gained popularity with the self-help book by Aron [4] and frequent mentions in the popular media. The psychometric evaluations of the Highly Sensitive Person Scale [5–7] have increased scientific research on SPS too. However, rather surprisingly, the role of SPS in the work environment remains unclear (but see [6, 8], for only two exceptions of studies on SPS in the work context). As approximately 20% of the people are said to score high on SPS [9], it is important to understand how SPS relates to employee health/well-being (e.g. burnout) and performance (e.g. extra-role behaviour), and whether employees characterised by high levels of SPS react differently to the work environment.

SPS has been linked to the phenomenon of differential susceptibility [10, 11]: people with high levels of SPS may react more strongly to both negative and positive stimuli. This implies that SPS may not only relate to a higher vulnerability to negative effects of adversity, but also to a disproportional susceptibility to the beneficial effects of benign situations [10]. Applying this reasoning to the context of work stress, we may expect employees with high levels of SPS to respond more strongly to both negative and positive work characteristics.

To study this, we turn to the Job Demands-Resources model (JD-R model; [12, 13]) offering an established framework on the effects of both negative (i.e. job demands) and positive work characteristics (i.e. job resources). We introduce SPS as a person-related factor in the JD-R model, acting both as a personal vulnerability factor and a personal resource that may moderate the relationship between work characteristics and employee health/well-being and performance, depending on the nature of those perceived work characteristics. More specifically, we predict that SPS acts as a personal vulnerability factor amplifying the relationship between job *demands* (i.e. workload and emotional demands) and emotional exhaustion (i.e. moderator of the energetic process). In addition, we hypothesise that SPS acts as a personal resource that strengthens the relationship between job *resources* (e.g. social support and learning opportunities) and proactive work behaviours such as helping one’s colleagues.

The current study contributes to the literature in several ways. First, this study goes beyond the evidence on the main effects of SPS presented by Evers, Rasche [6] and Lombard [8] by offering initial evidence on the moderating role of SPS in the relationship between work characteristics and employee health/well-being and performance. As such, it advances our understanding of why certain people react more strongly to the work environment than others. Second, it may further our understanding of both the negative and positive implications of SPS in the work context. By highlighting the possible beneficial implications of this trait, we may counter the rather negative treatment of SPS in previous studies in which SPS was mainly conceived as a vulnerability factor (e.g., [6, 8]). Third, we add SPS to the JD-R model. The idea that one trait acts as a personal vulnerability factor and a personal resource depending on the nature of the work characteristics experienced (job demands versus job resources) is quite new in the JD-R literature (see also [14]). Finally, this study may also have some important implications for practice. It may highlight that although investments in work stress prevention by decreasing job demands and increasing job resources are beneficial for all employees, they are particularly important to employees scoring high on SPS.

### Sensory-processing sensitivity and health

Although the concept of contextual sensitivity—referring to general sensitivity to contextual sensory information—was not new, in 1997, Aron and Aron [7] labelled this personality trait as *sensory-processing sensitivity* (SPS) and presented the Highly Sensitive Person Scale enabling scholars to scientifically measure SPS. Since then, this trait has attracted renewed interest. SPS is characterised by “involving deeper processing of stimuli across a very wide variety of situations, supported by a great response to both positive and negative stimuli that motivates learning and thus leads to more successful responses in future similar situations” ([1], p. 276). SPS thus has a clear advantage, although it comes at a cost in certain situations. People characterised by high levels of SPS—labelled as highly sensitive persons (HSPs)—are more sensitive to external sensory information including art, other’s moods, violence in the media, and being observed, as well as to internal stimuli such as caffeine, hunger and pain. As a consequence, HSPs are more easily over-aroused. Aron, Aron [1] describe four important (but incomplete) ways in which HSPs manage to be more responsive or sensitive: (1) inhibition of behaviour: especially in novel situations or situations with conflicting cues, HSPs may employ a responsive behavioural strategy (‘pause to check’) by taking time to evaluate environmental cues and to plan effective action; (2) greater awareness of subtle stimuli; (3) deeper processing of sensory information (either consciously or automatically); and (4) stronger emotional and stress reactions, including being easily aroused by too many stimuli. Although SPS has been linked to other personality traits such as introversion, emotionality and neuroticism, it has been found to differ from these traits [5, 7]. In this study, we treat SPS as a continuous construct rather than a dichotomous construct in which people are categorised as either HSPs or non-HSPs.

Whereas Aron and Aron [7] presented their Highly Sensitive Person Scale as a one-dimensional measurement tapping into individual differences in SPS, Smolewska, McCabe [5] identified three underlying dimensions (as confirmed in [6]). First, *ease of excitation* (EOE) refers to becoming mentally overwhelmed by external (e.g. change, other people’s moods, time pressure) and internal demands (e.g. hunger, pain). The second dimension, *aesthetic sensitivity* (AES), is characterised by an elevated awareness of aesthetic things such as art and music, subtleties and having a rich, complex inner life. Finally, *low sensory threshold* (LST) refers to an unpleasant sensory arousal to external stimuli; that is, being more easily overwhelmed by intense stimuli such as bright lights, strong smells, coarse fabrics or loud noises. Previous studies have identified specific patterns of associations for these components of SPS: while especially EOE and LST seem to relate to health complaints, AES is particularly related to outcomes that may be beneficial in social interactions and enhancing one’s complex inner life [15]. For instance, in the work context, Evers, Rasche [6] found that EOE and LST related to work displeasure and need for recovery, but AES was not related to these indicators of employee well-being. In a sample of psychology students, Liss, Mailloux [16] found that all three SPS components were positively related to anxiety, while only EOE and LST were related to greater depression. The components also seemed to relate differently to social outcomes: whereas EOE and LST were positively related to poor social skills, poor communication and difficulties describing emotions, AES was negatively related to poor communication. EOE and LST thus seem to be conceptually different from AES.

People with different levels of SPS may thus differ in the way they respond to both stressful and beneficial work environments in terms of health and well-being. In this respect, scholars have referred to the phenomenon of *differential susceptibility* [10, 11]. People differ in their susceptibility to environmental influences: some individuals may be disproportionately more vulnerable than others not only to the negative effects of destructive environmental stimuli, but may also be more susceptible to the positive effects of supporting and enriching experiences [10]. Belsky and Pluess [10] particularly introduce SPS as a marker of differential susceptibility (or a plasticity marker) that moderates the impact of environmental stimuli on individuals’ functions. This suggests that people with high levels of SPS may react more negatively when confronted with negative stimuli in comparison with people with low levels of SPS. At the same time, they may benefit more from positive stimuli. Several findings seem to provide support for these moderation effects of SPS [9]. Aron, Aron [17], for instance, found that SPS interacted with an adverse childhood environment to predict a greater state of negative affect across three independent studies. Similar results were found in a fourth study in which a negative experience was manipulated (i.e. carrying out difficult and frustrating ability tests). In addition, Pluess and Boniwell [18] found that a resilience-promoting programme reduced depressive symptoms in 11-year-old girls scoring high on SPS, but not in girls scoring low on SPS.

In the specific context of work stress, SPS may offer an explanation as to why certain employees react more strongly to both negative and positive work environments. It is therefore rather surprising that so little research has focused on the role of SPS in employees’ work experience. We are aware of only two studies on SPS in the work context [6, 8], both tapping into the direct relationships between SPS and perceived job characteristics (e.g. workload and emotional load) and indicators of employee well-being and performance (e.g. need for recovery, absenteeism). However, these studies focused mainly on SPS as a risk factor, neglecting possible advantages in the work context. These studies also only focused on the main effects, overlooking the possibility of a differential susceptibility among employees scoring high on SPS (SPS as a moderator in the stressor–strain relationship). We aimed to fill these gaps. We relied on the Job Demands-Resources model (JD-R model; [13]) to build our theoretical argumentation.

### Job demands-resources model and personal resources-vulnerability factors

The JD-R model [12, 13] divides job characteristics into job demands and job resources. Job demands—such as workload and emotional demands—are “those physical, psychological, social, or organizational aspects of the job that require sustained physical and/or psychological (cognitive and emotional) effort or skills and are therefore associated with certain physiological and/or psychological costs” ([13], p. 312). Job resources—such as task autonomy and social support—refer to “those physical, psychological, social, or organizational aspects of the job that help to either achieve work goals, reduce job demands and the associated physiological and psychological costs, or stimulate personal growth, learning, and development” ([12], p. 392).

The JD-R model distinguishes between two basic processes in which job demands and job resources may affect workers’ health and performance [12, 13]. First, in the *energetic or health impairment process*, job demands may consume employees’ mental and physical resources, leading to energy depletion or burnout (e.g., [19]). This may result in impaired health (e.g. anxiety, depression, and musculoskeletal and cardiovascular complaints) over time (e.g., [20]). Second, in the *motivational process*, the availability of job resources may foster workers’ willingness to put compensatory effort into the work task (extrinsic motivational role of job resources) and satisfy the basic psychological needs of autonomy, belongingness and competence (intrinsic motivational role of job resources) (e.g., [21, 22]). This in turn stimulates work engagement and other positive organisational outcomes such as employee performance [23]. While burnout specifically mediates the relationship between job demands and employee health, work engagement mediates the effect of job resources on positive organisational outcomes.

Next to job demands and job resources, scholars have integrated personal resources into the JD-R model [12, 24]. Personal resources—such as self-efficacy and optimism—refer to “psychological characteristics or aspects of the self that are generally associated with resiliency and that refer to the ability to control and impact one’s environment successfully” ([24], p. 49). Personal resources may intervene in the JD-R model in several ways, namely as antecedents of job demands and job resources, and as mediators or moderators of the relationships from job demands and job resources to health and performance [22, 24]. Regarding their moderating role, personal resources may buffer the effect of job demands on burnout, as they provide feelings of being in control of the demanding job situation and thus enable people to cope better with the demands of their job (e.g., [25]). In addition, personal resources may amplify the effect of job resources on work engagement, as they may help employees to mobilise their job resources (e.g., [26]).

When integrating personal factors into the JD-R model, scholars have mainly focused on positive factors or personal resources, overlooking the role of negative personal factors. It is only more recently that the integration of personal vulnerability factors (e.g. workaholism, neuroticism, pessimism)—also labelled as personal demands—into the JD-R model has been presented as a path for further research [24, 27]. Personal vulnerability factors may be defined as deficits in an individual’s physical, psychological and social resources that prevent them from warding off threats to something of importance (i.e., important choices, values and goals; see [28]). They may specifically increase the perception of job demands and make employees more vulnerable to the negative impact of job demands (e.g., amplifying the relationship between job demands and burnout; [27, 29]).

We want to draw on these theoretical principles of the JD-R model to formulate predictions on the interaction between SPS and the work environment in predicting employee reactions (heuristic use of the JD-R model; [24]), rather than to offer an exact and comprehensive test of the JD-R model [12, 13, 24]. Nevertheless, we contribute to the JD-R model by introducing SPS as a personality characteristic that may either act as a personal resource or a vulnerability factor, depending on the employee’s working situation in terms of job demands and resources.

### Current study

**Fig 1:**
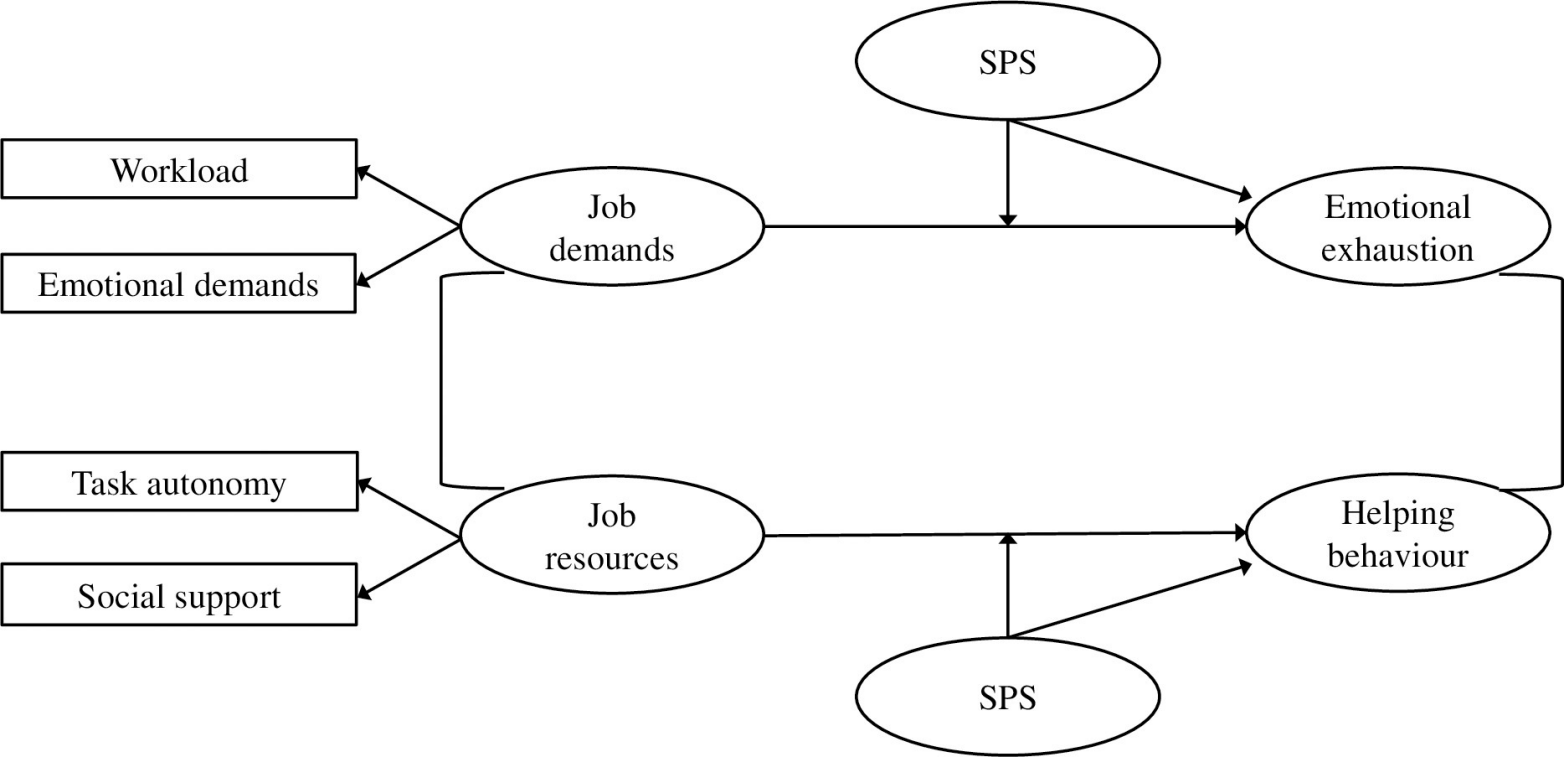
Theoretical model.

In this study, we aim to investigate the assumption that people with higher and lower SPS develop a differential susceptibility for the work environment characterised by job demands and resources. By integrating the literature on SPS [1, 10] and the JD-R model [13, 24], we suggest that, on the one hand, SPS functions as a personal vulnerability factor that strengthens the energetic or health impairment process. On the other hand, we expect SPS to act as a personal resource that strengthens the motivational process. Specifically, as displayed in Fig 1, we expect SPS to amplify the relationships between job demands (i.e. workload and emotional demands) and emotional exhaustion. Emotional exhaustion—referring to feelings of being emotionally drained by one’s job—is considered the central strain dimension of burnout [12], and is a typical outcome in the literature on SPS, as HSPs tend to invest more mental effort in each task due to deeper mental processing [30]. Furthermore, we expect SPS to amplify the relationship between job resources (i.e. social support and task autonomy) and helping behaviour. Helping behaviour—referring to acts of altruism, courtesy, peacekeeping and cheerleading—is a specific type of organisational citizenship behaviour or extra-role behaviour [31], and is selected as the outcome for the motivational process of the JD-R model. Helping behaviour is influenced by the motivational process as work engagement may free up resources because of increased efficiency, and positive emotions may stimulate social connection, resulting in behaviours that are not part of the job description but may be helpful to colleagues [12]. Moreover, based on the literature on SPS, HSPs pick up emotions and subtleties faster, leading to greater empathy and acts of helping behaviour [1].

Although we are not aware of previous studies investigating the amplifying role of SPS in the relationships of the JD-R model, indirect evidence comes from a study on another person-related factor both acting as a vulnerability factor and a personal resource. Lu, Lin [14] found that intrinsic work value orientation strengthened the negative relationship between work constraints and job satisfaction, as well as the positive relationship between work autonomy and job satisfaction. In line with our theoretical arguments and the research in the realm of SPS and the JD-R model, we thus predict:

*Hypothesis 1*: SPS moderates the relationship between job demands and emotional exhaustion, so that this relationship is stronger among employees scoring higher on SPS.
*Hypothesis 2*: SPS moderates the relationship between job resources and helping behaviour, so that this relationship is stronger among employees scoring higher on SPS.

To assess whether each SPS component (i.e. EOE, AES and LST) can account for the amplifying role of SPS in the relationships of the JD-R model, we investigate the hypotheses for each component separately (as recommended by [6, 15]).

## Method

### Participants

Data were collected between January 2014 and March 2015 among 2495 employees of nine organisations in Belgium. They were invited by IDEWE (a non-profit organisation advising employers on occupational health and safety) to participate in an assessment of psychosocial risks at their workplace. In total, 1411 employees filled out the questionnaire (response of 56.6%). We deleted all respondents with missing values on at least one of the study variables (see further under Measures), which resulted in a final sample of 1019 employees.

In this final sample, 67.6% were female and 59.7% were younger than 45 years old (categorical information about age could not be collected in two organisations and was available for only 816 respondents). The average tenure in the current position was 18.46 years (*SD* = 12.36). Next, the majority of the respondents (71.6%) had obtained a higher degree (Bachelor or Master), and 15.1% had a supervisory position (based on information from 1005 respondents). Furthermore, 70.3% of the respondents worked on a full-time basis (information about full-time versus part-time employment was available for 916 employees). Finally, seven of the nine organisations, comprising 766 respondents (75.2%), were private (rather than public) organisations. Three organisations were active in industry (*n* = 195; 19.1%); two in the service sector (*n* = 154, 15.1%); two in healthcare (*n* = 118; 11.6%); one in education (*n* = 191; 18.7%); and one was active in both healthcare and education (*n* = 361; 35.4%).

This project has been approved by the Committee for Medical Ethics (‘Commissie voor Medische Ethiek’) OG117 and was carried out according to the Belgian and international privacy and ethical legislation, allowing posthoc analyses of anonymised data obtained during occupational health surveillance and risk analysis.

### Measures

#### Job demands and resources

Workload, emotional demands and social support were measured using scales from the Short Inventory to Monitor Psychosocial Hazards (SIMPH) of Notelaers, De Witte [32]. The format of the items was adapted from questions to statements to increase the consistency of questioning throughout the survey (e.g. “Do you work under time constraints” was adapted to “I work under time constraints”). Item examples are “I work under time constraints” (workload, 3 items), “My work puts me in emotional situations” (emotional demands, 3 items) and “If necessary, I can ask my colleagues for help” (social support, 4 items). Task autonomy was measured using four items from Baillien, De Cuyper [33], for example, “I can plan my own work”. These items were rated on a five-point frequency scale ranging from 1 (*(almost) never*) to 5 (*(almost) always*). Cronbach’s alpha coefficients were .86, .87, .76 and .78 for workload, emotional demands, task autonomy and social support, respectively.

#### Outcomes

Emotional exhaustion was measured with the five-item subscale of the Utrecht Burnout Scale (UBOS-A) by Schaufeli and Van Dierendonck [34]. An item example is “I feel mentally exhausted because of my work”. The items were rated on a seven-point scale ranging from 1 (*never*) to 7 (*daily*). This scale had a Cronbach’s alpha coefficient of .90. Helping behaviour was measured by the seven-item subscale by Podsakoff [31] (e.g. “Help each other out if someone falls behind in his/her work”). The items were rated on a five-point scale ranging from 1 (*(almost) never*) to 5 (*(almost) always*). After deleting one item (based on the results of the CFAs: see below), the Cronbach’s alpha coefficient was .78.

**Sensory-processing sensitivity** (SPS) was measured with 23 items from the Highly Sensitive Person Scale by Aron and Aron [7]. The selection of 23 items of the original 27-item scale was based on Aron [35]. Again, the format of the items was adapted from questions to statements to increase the consistency of questioning throughout the survey (e.g. “Do other people’s moods affect you?” was adapted to “I’m affected by other people’s moods”). The items tapped into the three dimensions of SPS as found by Smolewska, McCabe [5], namely ease of excitation (EOE; 5 items; e.g. “I’m easily overwhelmed by things like bright lights, strong smells, coarse fabrics, or sirens close by”), aesthetic sensitivity (AES; 11 items; e.g. “I’m affected by other people’s moods”) and low sensory threshold (LST; 7 items; e.g. “I’m deeply moved by art or music”). The items were rated on a five-point Likert scale ranging from 1 (*entirely disagree*) to 5 (*entirely agree*). Cronbach’s alpha coefficients for the final EOE, AES and LST scales were .80, .69 and .66, respectively (after deleting certain items based on the CFAs: see below).

#### Covariates

Gender (female = 0; male = 1), occupational tenure (years), education (1 = primary education; 1^st^ to 6^th^ grade, 2 = first level of secondary education; 7^th^ to 9^th^ grade, 3 = second level of secondary education; starting from 10^th^ grade, 4 = Bachelor degree, 5 = Master degree) and sector (public sector = 0; private sector = 1) were used as covariates. Hypotheses were tested with and without controlling for these covariates. As both types of analyses led to the same conclusions, but including covariates came at the cost of the parsimoniousness and the fit of the model, we will only describe the results from the analyses without covariates.

### Statistical analyses

Hypotheses were tested using Structural Equation Modelling (Maximum Likelihood estimation with Robust standard errors; MLR) by means of MPlus 7.11 [36]. First, a series of confirmatory factor analyses (CFA) were conducted to evaluate the hypothesised measurement model; a seven-factor measurement model with job demands, job resources, EOE, AES, LST, emotional exhaustion and helping behaviour as the latent factors. While EOE, AES, LST, emotional exhaustion and helping behaviour were measured by their respective items, the latent factors of job demands and resources were regressed on the manifest scales of workload and emotional demands, and of autonomy and social support, respectively. The hypothesised measurement model was compared with alternative factor models to examine the dimensionality of the study scales.

Second, several structural models were tested and compared. In a first step, we tested a main effect model with structural paths from job demands and the three dimensions of SPS to emotional exhaustion, and from job resources and the three dimensions of SPS to helping behaviour. In this model, we also controlled for the cross-paths from job demands to helping behaviour and from job resources to emotional exhaustion. The latent factors job demands and job resources were allowed to co-vary, as were the three dimensions of SPS. In a second step, we added one of each of the interactions (i.e. job demands*EOE → emotional exhaustion, job demands*AES → emotional exhaustion, job demands*LST → emotional exhaustion, job resources*EOE → helping behaviour, job resources*AES → helping behaviour, job resources*LST → helping behaviour) to the main effect model. The interactions were tested in separate models, as we estimated interactions between latent factors (a highly intensive estimation procedure).

Model fit was evaluated based on the Comparative Fit Index (CFI), the Non-Normed Fit Index (NNFI), the Root Mean Square Error of Approximation (RMSEA) and the Standardised Root Mean square Residual (SRMR). Values of CFI and NNFI equal to or larger than .90, and values of RMSEA and SRMR from .08 and .10, respectively, indicate a good fit. Nested models were compared using the *Chi*^*2*^ or the −2LL difference test.

## Results

### Descriptive findings

Table 1 displays the means, standard deviations and the inter-correlations for the study scales.

**Table 1:** Means, standard deviations, cronbach’s alpha coefficients and correlation matrix (*N* = 1019).

Variable	*M*	*SD*	1	2	3	4	5	6	7	8	9
**1. Workload**	3.36	0.81	(.85)	.32***	-.20***	-.21***	.14***	.10**	.12***	.40***	.22***
**2. Emotional demands**	2.89	0.91		(.87)	-.03	-.10**	.20***	.24***	.17***	.31***	.25***
**3. Task autonomy**	3.47	0.78			(.77)	.33***	.02	.03	-.07*	-.24***	.05
**4. Social support**	3.85	0.81				(.79)	-.04	.04	-.12***	-.31***	.14***
**5. Ease of excitation (EOE)**	2.98	0.66					(.80)	.30***	.47***	.29***	.04
**6. Aesthetic Sensitivity (AES)**	3.33	0.78						(.69)	.39***	.09**	.23***
**7. Low sensory threshold (LST)**	2.17	0.88							(.66)	.23***	.08*
**8. Emotional exhaustion**	2.84	1.36								(.90)	.06
**9. Helping behaviour**	3.49	0.61									(.78)

* p < .05

** p < .01

*** p < .001.

### Test of the measurement model

Before testing the hypotheses, we carefully inspected our measurement model based on a series of CFAs, as summarised in Table 2. However, the initial hypothesised measurement model did not fit the data well. The results showed potential cross-loadings and low factor loadings for multiple items (mostly SPS items). So, we followed a strict stepwise procedure in which we first omitted items with cross-loadings (based on modification indices) and items with factor loadings below .40 [37]. In total, seven items were dropped for further analysis (one item of the helping behaviour scale due to small factor loading: “Willingly share my expertise with other members of the crew”; and six SPS items due to cross-loadings and small factor loadings), resulting in a good measurement model without problematic items (CFI = .92; NNFI = .91, RMSEA = .04; SRMR = .04). Table 3 gives an overview of all SPS item descriptions and the factor loadings of the items in the final reduced measurement model. We refer to Aron and Aron [7] for an overview of the original items.

**Table 2:** Results of the item analysis and confirmatory factor analysis (*N* = 1019).

Model	Latent factors	Omitted indicator	*Chi*^*2*^(*df*)	CFI	NNFI	RMSEA	SRMR	Compared model	Δ*Chi*^*2*^(*df*)
1. Full hypothesised measurement model	Dem., Res., Exh., Help, EOE, AES, LST	–	2359.87(681)***	.834	.819	.049	.059	–	–
2. Reduced hypothesised measurement model 1	Dem., Res., Exh., Help, EOE, AES, LST	SPS5	1933.55(644)***	.861	.848	.045	.047	Model 1	366.32(37)***
3. Reduced hypothesised measurement model 2	Dem., Res., Exh., Help, EOE, AES, LST	SPS19	1792.90(608)***	.872	.860	.044	.045	Model 2	140.65(36)***
4. Reduced hypothesised measurement model 3	Dem., Res., Exh., Help, EOE, AES, LST	SPS17	1674.16(573)***	.879	.867	.043	.044	Model 3	118.74(35)***
5. Reduced hypothesised measurement model 4	Dem., Res., Exh., Help, EOE, AES, LST	Help2	1458.56(539)***	.896	.885	.041	.043	Model 4	215.60(34)***
6. Reduced hypothesised measurement model 5	Dem., Res., Exh., Help, EOE, AES, LST	SPS2	1342.40(506)***	.904	.893	.040	.042	Model 5	116.16(33)***
7. Reduced hypothesised measurement model 6	Dem., Res., Exh., Help, EOE, AES, LST	SPS18	1203.03(474)***	.914	.904	.039	.040	Model 6	139.37(32)***
**8. Final reduced hypothesised measurement model**	**Dem., Res., Exh., Help, EOE, AES, LST**	**SPS15**	1148.44(443)***	**.916**	**.906**	**.040**	**.040**	**Model 7**	54.59(31)***
9. Six-factor model 1	Job characteristics (Dem. + Res.), Exh., Help, EOE, AES, LST	–	1330.92(449)***	.895	.884	.044	.049	Model 8	182.48(6)***
10. Six-factor model 2	Dem., Res., Outcome (Exh. + Help), EOE, AES, LST	–	2447.32(449)***	.762	.737	.066	.079	Model 8	1298.88(6)***
11. Five-factor model	Dem., Res., Exh., Help, SPS (EOE + AES + LST)	–	1899.22(454)***	.828	.812	.056	.057	Model 8	750.78(11)***
12. One-factor model	General factor	–	5325.53(464)***	.421	.381	.101	.121	Model 8	4177.09(21)***

Dem. = Job demands; Res. = Job Resources; Exh. = Emotional exhaustion; Help = Helping behaviour; EOE = ease of excitation; AES = Aesthetic sensitivity; LST = low sensory threshold; Job = job characteristics; CFI = comparative fit index; NNFI = non-normed fit index; RMSEA = root mean square error of approximation; SRMR = standardised root mean square residual.

*p < .05

**p < .01

***p < .001.

**Table 3:** Overview of the SPS subscales and their standardised factor loadings in the final reduced hypothesised measurement model.

Item Number	Item Description	Latent factors		
		EOE	AES	LST
3	Affected by other people’s moods	.486		
4	Sensitive to pain	.463		
13	Startling easily	.532		
14	Rattled under time pressure	.703		
16	Annoyed by people putting pressure on me	.640		
20	Strong reaction when being hungry	.448		
21	Shaken up by changes in life	.620		
24	Avoiding upsetting or overwhelming situations	.506		
26	Nervous when competing/being observed while performing a task	.583		
27	Sensitive or shy as a child	.438		
8	Rich inner life		.599	
10	Moved by arts/music		.704	
12	Conscientious		.495	
22	Noticing and enjoying delicate things		.616	
6	Sensitive to caffeine			.498
7	Overwhelmed by intense external stimuli			.748
9	Uncomfortable by loud noise			.697
**Omitted items:**		**Dimension following Smolewska, McCabe [5]:**		
17	Avoiding mistakes/forgetting things	X		
2	Awareness of environmental subtleties		X	
5	Withdrawing during busy days		X	
15	Making people comfortable by adjusting the physical environment		X	
18	Avoiding violent movies/TV shows			X
19	Unpleasant arousal			X

**Note.** The items derive from the Highly Sensitive Person Scale of Aron and Aron [7]. The classification of the items into the dimensions of Ease of Excitation (EOE), Aesthetic Sensitivity (AES) and Low Sensory Threshold (LST) was based on Smolewska, McCabe [5].

Furthermore, the final reduced hypothesised measurement model was compared and showed a better fit than four alternative measurement models (as summarised in the lower part of Table 2): (1) a six-factor model in which the job demands and resources were taken together (Δ*Chi*^2^(6) = 182.48, *p* < .001), (2) a six-factor model in which the items of the emotional exhaustion and helping behaviour scales loaded on a general outcome factor (Δ*Chi*^2^(6) = 1298.88, *p* < .001), (3) a five-factor model with a general SPS factor (Δ*Chi*^2^(11) = 750.78, *p* < .001), and (4) a one-factor model (Δ*Chi*^2^(21) = 4177.09, *p* < .001). These results thus demonstrate the expected dimensionality of the study variables.

### Test of the hypotheses

The findings of the SEM analyses are displayed in Table 4. The results of the main effect model show that job demands were positively related to emotional exhaustion, while the job resources were positively associated with helping behaviour. Next, EOE was positively associated with emotional exhaustion and negatively with helping behaviour. AES, however, was negatively related to emotional exhaustion. We did not find a direct relationship between AES and helping behaviour, and between LST and both outcomes.

**Table 4:** Results of the structural equation modelling analyses (*N* = 1019).

Model	(Added) structural paths	Standardised ß-coefficient	Unstandardised B-coefficient (SE)	–2LL(*df*)	CFI	NNFI	RMSEA	SRMR	Compared model	Δ–2LL(*df*)
1. Main effect model	Dem. → Exh.	.55***	1.45(.25)***							
	Res. → Exh.	–.24**	–0.81(.24)**							
	EOE → Exh.	.15*	0.39(.17)*							
	AES → Exh.	–.14*	–0.30(.13)*							
	LST → Exh.	.07	0.15(.15)							
	Dem. → Help	.69***	0.72(.02)***							
	Res. → Help	.49***	0.66(.15)***							
	EOE → Help	–.24**	–0.26(.09)**							
	AES → Help	.10	0.08(.07)							
	LST → Help	.07	0.07(.08)	88842.14(117)	.916	.906	.040	.040	–	–
2. Interaction model 1	(+) Dem. * EOE → Exh.	na	0.60(.21)**	88830.48(118)	na	na	na	na	Model 1	11.66(1)***
3. Interaction model 2	(+) Dem. * AES → Exh.	na	0.15(.21)	88841.11(118)	na	na	na	na	Model 1	1.03(1)
4. Interaction model 3	(+) Dem. * LST → Exh.	na	0.62(.20)**	88828.16(118)	na	na	na	na	Model 1	13.98(1)***
5. Interaction model 4	(+) Res. * EOE → Help	na	0.24(.25)	88839.65(118)	na	na	na	na	Model 1	2.49(1)
6. Interaction model 5	(+) Res. * AES → Help	na	–0.02(.15)	88842.10(118)	na	na	na	na	Model 1	0.04(1)
7. Interaction model 6	(+) Res. * LST → Help	na	0.38(.16)*	88830.72(118)	na	na	na	na	Model 1	11.42(1)***

*p < .05

**p < .01

***p < .001.

**Fig 2:**
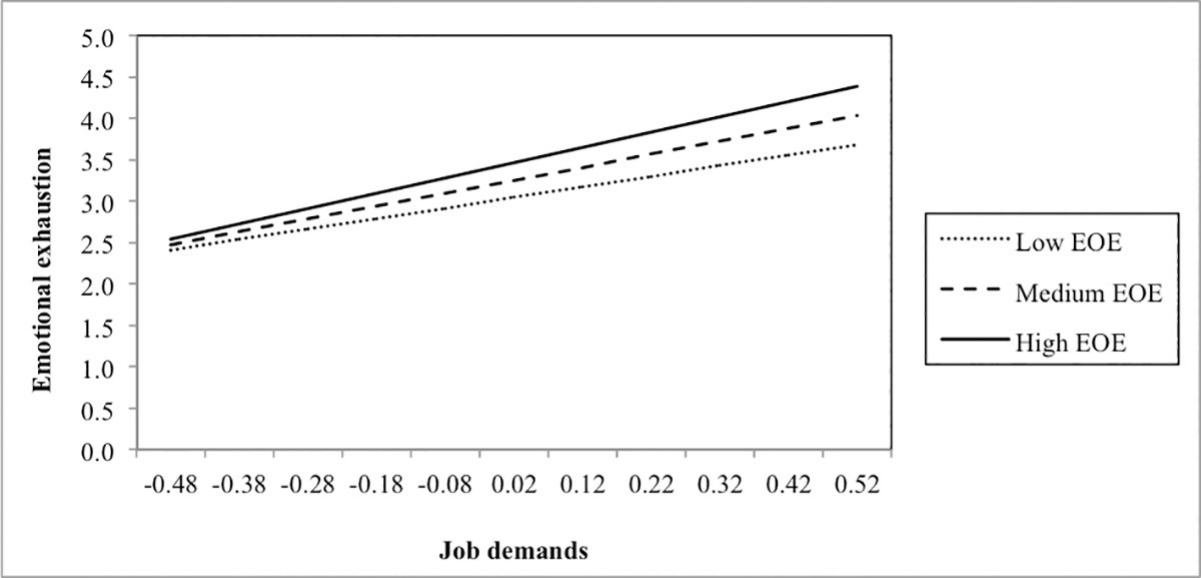
Interaction between job demands and ease of excitation (EOE) in predicting emotional exhaustion.

**Fig 3:**
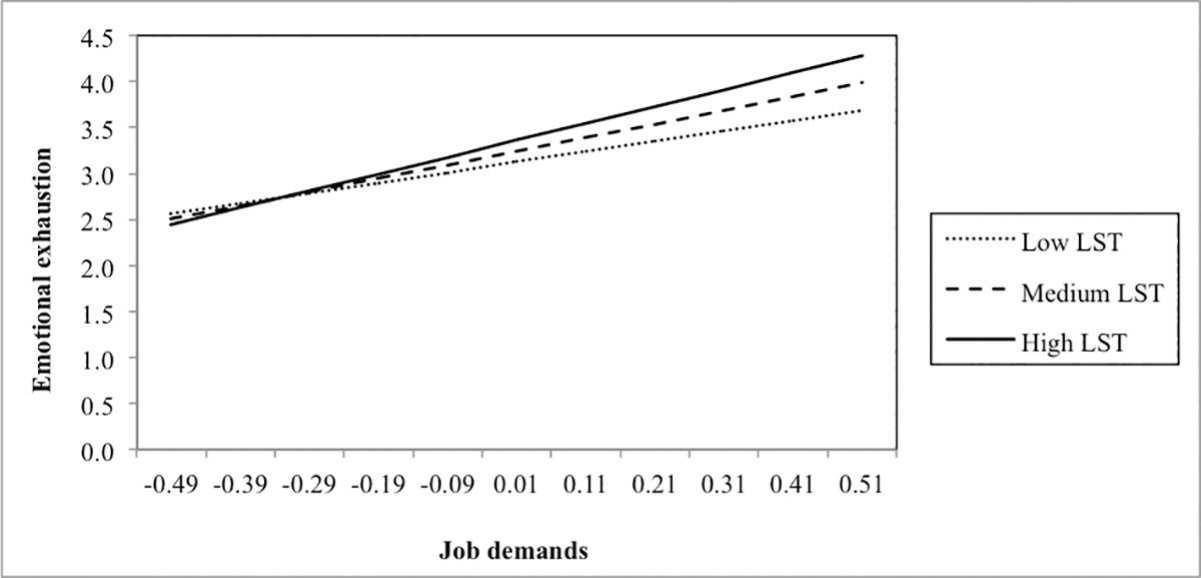
Interaction between job demands and low sensory threshold (LST) in predicting emotional exhaustion.

**Fig 4:**
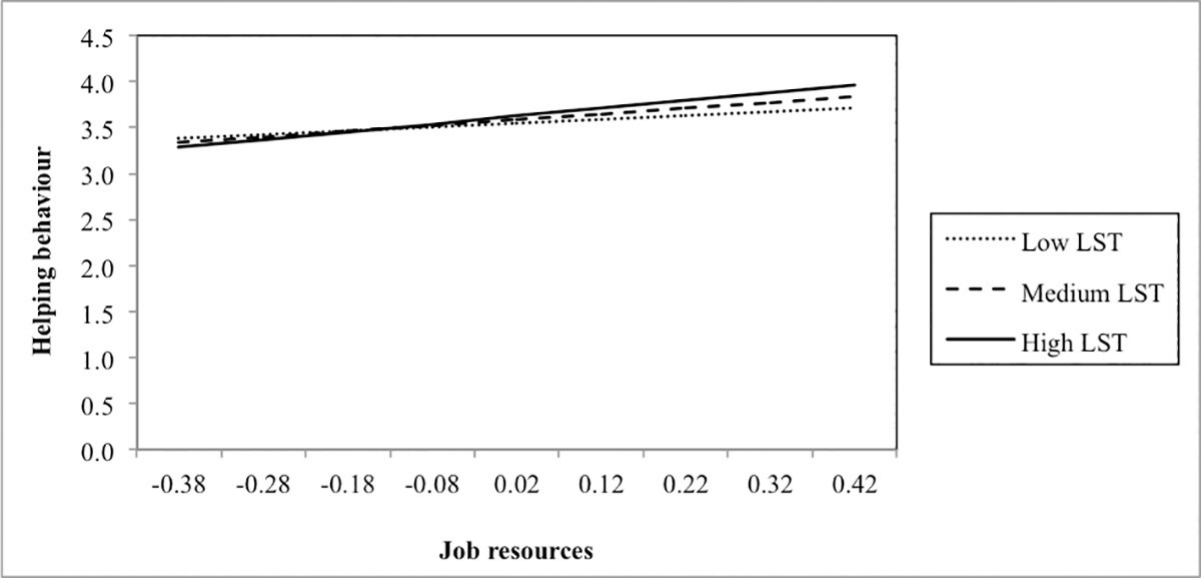
Interaction between job resources and low sensory threshold (LST) in predicting helping behaviour.

In addition, we added single interaction terms to the main effect model (see lower part of Table 4). Three significant interactions were found. First, the interaction between job demands and EOE was positively related to emotional exhaustion. Adding this interaction term to the main effect model significantly increased model fit. Plotting this interaction (Fig 2) showed that EOE acted as an amplifier of the relationship between job demands and emotional exhaustion: job demands were more negatively related to emotional exhaustion among respondents scoring higher on EOE (slopes at different values of EOE: –1 *SD*: B = 1.28, *SE* = .25, *p* < .001; +1 *SD*: B = 1.85, *SE* = .31, *p* < .001). Second, a similar result was found for LST: LST amplified the relationship between job demands and emotional exhaustion, meaning that job demands were more strongly related to emotional exhaustion among persons scoring higher on LST (slopes at different values of LST: –1 *SD*: B = 1.13, *SE* = .25, *p* < .001; +1 *SD*: B = 1.83, *SE* = .29, *p* < .001) (Fig 3). Finally, we also found a significant moderation effect of LST in the relationship between job resources and helping behaviour. Fig 4 shows that job resources were more strongly positively related to helping behaviour among respondents scoring higher on LST (slopes at different values of LST: –1 *SD*: B = .41, *SE* = .18, *p* < .05; +1 *SD*: B = 0.84, *SE* = .16, *p* < .001).

## Discussion

The aim of this study was to investigate the principle of differential susceptibility to cues in the work environment [10, 11] of people scoring higher rather than lower on SPS. Based on the literature on SPS [1, 7] and the JD-R model [12, 13, 24], we predicted that SPS acts as a vulnerability factor, amplifying the relationship between job demands and emotional exhaustion. At the same time, it may act as a personal resource increasing the relationship between job resources and helping behaviour. These predictions were investigated for each of the three dimensions of SPS (i.e. EOE, AES and LST) separately, in line with previous recommendations [6, 15]. The results offered first evidence for the greater susceptibility of persons with higher levels of SPS to the work context: EOE and LST amplified the positive relationship between job demands and emotional exhaustion, and LST also amplified the positive relationship between job resources and helping behaviour.

We found support for three out of six moderation effects. First, the amplifying effect of EOE and LST in the positive relationship between job demands and emotional exhaustion demonstrates that these components of SPS can be considered as personal vulnerability factors. In work situations with greater job demands (i.e. workload and emotional demands), employees scoring higher on EOE and LST experienced more emotional exhaustion, compared to employees scoring lower on EOE and LST. The idea that SPS can be considered as a strain-enhancing factor is not new and dominates the majority of previous studies on this topic (e.g., [6]). Highly sensitive persons are more sensitive to external sensory information, and as a consequence they are more easily over-aroused [1]. Therefore, they may show stronger emotional and stress reactions to intense stimuli, such as high job demands. In particular, the components of EOE and LST could relate to the increased sensitivity to *negative* external cues (e.g. job demands), which might explain why we only found evidence for an amplifying effect of the job demands–emotional exhaustion relationship for these components and not for AES. Although we offered initial evidence in the work context, our results align with previous findings [9]. Aron, Aron [17], for instance, found that SPS interacted with an adverse childhood environment as well as with a manipulated negative experience (i.e. carrying out difficult and frustrating ability tests) in predicting a greater state of negative affect.

Second, the amplifying effect of LST in the positive relationship between job resources and helping behaviour demonstrates that LST may also act as a personal resource. In work situations with greater job resources (e.g. task autonomy and social support), employees scoring higher (in comparison with lower) on LST expressed more helping behaviour. Under optimal conditions (e.g. work situations with many job resources), highly sensitive persons may not be as preoccupied with processing sensory information and may fully respond to these supporting and enriching experiences, to which they are more susceptible [1]. Their greater awareness of subtleties, and of social cues, may then result in more prosocial behaviours such as helping behaviour. In this study, particularly the items of the scale LST seem to tap into the greater *awareness* of environmental subtleties and this might explain why we found LST to amplify the relationship between job resources and helping behaviour. Only a few previous studies have focused on the moderating effect of SPS on the relationship between positive environmental stimuli and prosocial behaviour (e.g., [38]). Future research should therefore replicate our findings.

The current study not only contributed to the literature on SPS—by enhancing our knowledge of its moderating role in the relationship between work characteristics and employee health/well-being and performance—, it also added to the JD-R model [12, 13]. Based on our results, we may introduce SPS into the JD-R model both as a personal vulnerability factor and a personal resource. Employees scoring higher on SPS may react more negatively to job demands and more positively to job resources in terms of health/well-being and performance. Faced with greater job demands, highly sensitive persons may feel overwhelmed and may therefore lack the resources to deal with those demands (cfr. definition of a personal vulnerability factor), resulting in higher levels of strain. However, in work situations with many job resources, highly sensitive persons may feel resilient, in control and able to successfully respond to environmental cues (cfr. definition of a personal resource), resulting in more helping behaviour. By presenting the same trait as a personal vulnerability factor *and* a personal resource, we proceed with a highly innovative, but more nuanced avenue in the research on the JD-R model (along with [14]).

Although not the focus of this study, we also examined direct relationships between the SPS components and emotional exhaustion and helping behaviour when calculating bivariate correlations and when testing our SEM models. The results of both types of analyses differed, however. The correlations showed that all three SPS components related positively to emotional exhaustion, and that AES and LST were positively associated with helping behaviour (see Table 1). Following the SEM analyses (see Table 4), EOE was positively related to emotional exhaustion and negatively to helping behaviour, and AES was negatively related to emotional exhaustion. While previous studies focusing on the relationship between the SPS components and well-being have chiefly relied on the results of correlations (e.g., [6, 15]), we conducted SEM analysis as this technique has important advantages [39]. In addition to modelling measurement error and calculating model fit, SEM analysis allows for the testing of all hypothesised relationships simultaneously in one structural model, implying that the effect of each SPS component was tested while controlling for the effects of the other SPS components. This might explain the different results for the correlations and the SEM analysis: the relationships of the SPS components with other variables might have changed when controlling for the other SPS components because parts of the SPS measures are redundant. Nevertheless, in this study, we decided to treat the SPS components as separate latent factors, as the results demonstrated theoretically meaningful differences in the relationships of the SPS components (see also [6, 15]). Moreover, the inter-correlations between the SPS components were of medium size (.30 < *r* < .50; [40]), demonstrating that multicollinearity (bivariate correlations higher than *r* = .85; [39]) might not be a major concern.

### Study limitations and paths for future research

As in any other study, our present study yields several limitations and leads for the future that need to be addressed. First, we relied on self-reports for all study variables. Consequently, our findings could have been influenced by common method bias. However, as common method variance rather deflates than inflates interaction effects, we do not expect our conclusions regarding the hypotheses to be significantly impacted by this method bias [41]. Furthermore, in our study design, we invested in recommended actions in order to prevent this bias [41]. For example, we guaranteed confidentiality of the information gathered through the questionnaire and underlined that there were no right or wrong answers. While our findings are informative as one of the first explorations of SPS in the work context, future research could nevertheless add to this line of research by re-testing our hypotheses using more objective measures of environmental sensitivity. Examples could be multi-source data provided by experts in SPS diagnosis or experimental designs in which participants scoring high versus low on SPS are confronted with a range of stressors and resources.

Second, our study showed a rather low internal consistency for AES and LST and required that many items were omitted from the SPS subscales to improve model fit. In all, this indicates that the psychometric properties of the three subscales of the Highly Sensitive Person Scale are questionable. Notably, whereas our study was conducted among adults, most studies on the psychometric properties of the Highly Sensitive Person Scale were performed in adolescents and young adults [9]. More research is required to improve the adult SPS scale.

Third, we relied on cross-sectional data and any conclusion regarding causality should be handled with care. As such, building our study around the well-established JD-R model—which has been extensively tested and validated with longitudinal designs—contains a particular strength of the current study. Future studies could, however, investigate our hypothesis using multiple waves, allowing for a growing insight into the exact role of SPS in the work context, also over time.

### Implications for practice

Our research further substantiated the results from an abundance of studies on the JD-R model [12, 13] demonstrating the positive relationship between job demands and emotional exhaustion, and between job resources and positive behavioural outcomes such as helping behaviour. As such, practitioners aiming to increase well-being among the organisation’s staff by preventing exhaustion and stimulating helping behaviour may feel confident in using the JD-R model’s distinction between job demands and job resources as a valuable lens through which to interpret the work environment. A risk assessment plotting employees’ exposure to job demands and job resources could inform the practitioner and organisation about the presence of high demands or a lack of resources, and reveal leads for effective stress prevention and well-being promotion by decreasing demands and increasing resources.

Additionally, our findings underscore that, while stress prevention and well-being promotion is beneficial for all employees, this may be particularly so for employees scoring high on SPS. Specifically, we found that EOE and LST amplified the positive relationship between job demands and emotional exhaustion. Consequently, especially for high SPS employees, a decrease in job demands will be associated with lower levels of exhaustion and thus higher well-being. Alternatively, we found that LST amplified the positive job resources–helping behaviour relationship. This suggests that higher levels of job resources relate to higher levels of helping behaviour at work, particularly for employees scoring high on SPS. As such, a well thought-out prevention programme following the JD-R model’s premises on job resources raises the added value of having high SPS employees in the organisation as these employees in particular will contribute to positive well-being and to desired helping behaviour.

## Conclusions

Overall, the results of this study suggest that—depending on the nature of the work environment in terms of job demands–resources—SPS can be conceived as a personal vulnerability factor and a personal resource that boosts the energetic and motivational process, as outlined in the JD-R model. While EOE and LST were found to amplify the relationship between job demands (i.e. workload and emotional demands) and emotional exhaustion, only LST amplified the relationship between job resources (i.e. task autonomy and social support) and helping behaviour. This study expands our knowledge by providing evidence of the phenomenon of differential susceptibility of highly sensitive persons to their work environment, and contributes to the JD-R model by adding SPS as a new moderating trait or person-related variable to the model.

## Supporting information

S1 Data Data file. Data set, including raw data on the study measures, that was used to conduct the Confirmatory Factor Analyses and the Structural Equation Modelling analyses.
(CSV)
